# Beneficial Effects of Sulforaphane Treatment in Alzheimer's Disease May Be Mediated through Reduced HDAC1/3 and Increased P75NTR Expression

**DOI:** 10.3389/fnagi.2017.00121

**Published:** 2017-05-01

**Authors:** Jingzhu Zhang, Rui Zhang, Zhipeng Zhan, Xinhui Li, Fuyuan Zhou, Aiping Xing, Congmin Jiang, Yanqiu Chen, Li An

**Affiliations:** ^1^School of Public Health, China Medical UniversityShenyang, China; ^2^Ningxia Key Laboratory of Cerebrocranial Diseases, School of Laboratory Medicine, Ningxia Medical UniversityYinchuan, China

**Keywords:** Alzheimer's disease, amyloid-β, sulforaphane, p75 neurotrophin receptor, histone deacetylases

## Abstract

Alzheimer's disease is an irreversible, progressive neurodegenerative disorder. The accumulation of Aβ in the brain is thought to play a causative role in the development of cognitive dysfunction in Alzheimer's disease. The p75 neurotrophin receptor is of great importance to protect against the Aβ burden and its expression is regulated by histone acetylation. This study investigated whether the phytochemical sulforaphane, a pan-histone deacetylase inhibitor, up-regulates the p75 neurotrophin receptor expression via affecting histone acetylation in protection against Alzheimer's disease. We found that sulforaphane ameliorated behavioral cognitive impairments and attenuated brain Aβ burden in Alzheimer's disease model mice. Additionally, sulforaphane reduced the expression of histone deacetylase1, 2, and 3, up-regulated p75 neurotrophin receptor, and increased levels of acetylated histone 3 lysine 9 and acetylated histone 4 lysine 12 in the cerebral cortex of Alzheimer's disease model mice as well as in Aβ-exposed SH-SY5Y cells. Furthermore, silencing of histone deacetylase1 and 3, but not histone deacetylase2, gene expression with small interfering RNA caused up-regulation of p75 neurotrophin receptor in SH-SY5Y cells. In conclusion, this study demonstrates that sulforaphane can ameliorate neurobehavioral deficits and reduce the Aβ burden in Alzheimer's disease model mice, and the mechanism underlying these effects may be associated with up-regulation of p75 neurotrophin receptor mediated, apparently at least in part, via reducing the expression of histone deacetylase1 and 3.

## Introduction

Alzheimer's disease (AD) is an irreversible, progressive neurodegenerative disorder that affects more than 46 million people worldwide (Prince, 2015). The economic impact of AD on families and society presents a major challenge to public health. Recent memory impairment is the earliest notable symptom of patients with AD, and this symptom progresses gradually into severe dementia, characterized by impairments in learning, memory retrieval, reasoning, communication, and one's ability to carry out daily activities (Chin-Chan et al., 2015). Morphologically, AD is characterized by extracellular deposition of amyloid-beta (Aβ) protein combined with formation of senile plaques, intracellular neurofibrillary tangles (NFTs), and the death of cholinergic neurons (Attems et al., 2012). The “Aβ cascade hypothesis” postulates that the fundamental pathogenetic mechanism responsible for neuronal degenerative changes and the compromise of cognitive functions in AD is an excessive accumulation of Aβ in the brain (Hardy and Selkoe, 2002). Accordingly, reducing the brain Aβ burden has become a key strategy in AD therapy and prevention.

The p75 neurotrophin receptor (p75NTR) is a single membrane spanning protein in the tumor necrosis factor (TNF) receptor family. Signaling by p75NTR has been implicated in diverse neuronal responses. Several lines of evidence indicate that p75NTR plays a role in critical hallmarks of AD, including Aβ production and deposition, neuronal death, neurite degeneration, tau phosphorylation, cell cycle re-entry, and cognitive impairment (Zeng et al., 2011). Several studies have shown significantly reduced expression of p75NTR in the brains of AD patients (Kordower et al., 1989; Arendt et al., 1997; Salehi et al., 2000). Likewise, levels of the p75NTR-ectodomain—the ectodomain shedding of p75NTR that protects neurons against Aβ toxicity—have been shown to be reduced in the brains of AD patients and an AD mouse model (APP/PS1 double-transgenic mice; Yao et al., 2015). In addition, insoluble Aβ deposition has been reported to be elevated in the brain after deletion of the p75NTR-ectodomain gene in APP/PS1 double-transgenic mice, and intra-hippocampal injection of p75NTR-ectodomain has been shown to reduce local Aβ plaques (Wang et al., 2011). These findings suggest that up-regulation of p75NTR expression may be a therapeutic target for reducing the brain Aβ burden in AD.

The etiology of AD has been shown to be affected by epigenetic regulation via acetylation of histones on their amino-terminus lysine residues, specifically acetylation of histone 3 lysine 9 (H3K9) and H4K12 (Walker et al., 2013; Plagg et al., 2015). As we know, histone acetylation status is modified by actions of histone acetyltransferases (HATs) and histone deacetylases (HDACs). In mammals, HDACs are divided into four groups: the zinc-dependent class I (HDAC1–3 and 8), II (HDAC4–7, 9, and 10) and IV (HDAC11) HDACs, and the NAD^+^-dependent class III HDACs (SIRT1–7; members of the sirtuin protein family). Emerging evidence has implicated that HDACs may be potential therapeutical targets for the treatment of AD (Xu et al., 2011). A study on neuroblastoma malignancy development showed that p75NTR expression was repressed when HDAC1 bound to its promoter and that this effect was blocked in the presence of the pan-HDAC inhibitor trichostatin A (TSA) (Iraci et al., 2011). However, it is not known whether HDAC1 plays a role in the regulation of p75NTR transcription by altering histone acetylation in AD. Moreover, HDAC2 and HDAC3, other two class I HDACs, have been found to be implicated in AD (Xu et al., 2011). What is more important, it has not yet been documented whether HDAC2/HDAC3 participates in regulating the expression of p75NTR in AD.

There is great interest in identifying plant metabolites with medicinal properties, including for the prevention and treatment of neurodegenerative disorders. Sulforaphane (SFN) is a secondary metabolite found in edible cruciferous vegetables that has been described as an anti-oxidant (Zhang et al., 2015) or an anti-inflammatory agent (Brandenburg et al., 2010) and has been reported to have potent neuroprotective effects. Because SFN also acts as a pan-HDAC inhibitor, suppressing not only HDAC activity but also the expression of class I and class II HDACs (Dashwood and Ho, 2007; Su et al., 2014), it is possible that SFN may exert a protective effect against AD by way of HDAC inhibition.

In this study, we found that the beneficial effects of SFN treatment in AD may be associated with up-regulation of p75NTR expression, which is mediated, at least partly, by SFN-induced reduction of HDAC1 and HDAC3 expression.

## Materials and methods

### Reagents

D, L-SFN (purity ≥ 97.0%) was purchased from Toronto Research Chemicals, Inc. (Toronto, Canada). Detailed information about the primary antibodies used is presented in Table 1. All secondary antibodies were from Beijing Zhongshan Biotechnology (Beijing, China). Cell culture medium was obtained from American Hyclone Inc. (USA). Aβ_25−35_ (toxic fragment of the full-length Aβ peptide; American Peptide Inc., USA) was solubilized in sterile water at 1 mM concentration and aggregated by *in vitro* incubation at 37°C for 7 days. Immunohistochemistry kits were purchased from Beijing Zhongshan Biotechnology (Beijing, China).

**Table 1:** **Details of primary antibodies**.

**Antibody**	**Dilution**	**Company**	**Catalog number**	**Antibody**	**Dilution**	**Company**	**Catalog number**
Rabbit anti-Aβ_1−42_ polyclonal antibody	1:200	Abcam Inc.	ab10148	Rabbit anti-HDAC1 polyclonal antibody	1:1000	Santa Cruz Biotechnology	sc-7872
Rabbit anti-p75NTR polyclonal antibody	1:1500	Abcam Inc.	ab8874	Rabbit anti-HDAC2 polyclonal antibody	1:1000	Santa Cruz Biotechnology	sc-7899
Rabbit anti-Ace-H3K9 polyclonal antibody	1:200	ImmunoWay Inc.	YK0006	Rabbit anti-HDAC3 polyclonal antibody	1:1000	Santa Cruz Biotechnology	sc-11417
Rabbit anti-Ace-H4K12 polyclonal antibody	1:200	ImmunoWay Inc.	YK0013	Rabbit anti-β-actin polyclonal antibody	1:1000	Santa Cruz Biotechnology	sc-130656
Rabbit anti-Histone H3.1 polyclonal antibody	1:200	ImmunoWay Inc.	YK0009				

### *In vivo* study

#### Animals and treatment

We purchased APP/PS1 double-transgenic mice [B6C3-Tg (APPswe, PS1dE) 85Dbo/J] and wild-type littermates from Jackson Laboratory (USA). APP/PS1 mice develop Aβ deposits in brain by 6–7 months of age. The mice were housed in a temperature (22 ± 2°C), humidity (55 ± 15%) controlled environment, maintained on a 12-h light-dark cycle, and given access to food and water *ad libitum*. The animal experiment was approved by the Animal Care and Use Committee of China Medical University, which complies with the National Institutes of Health Guide for the Care and Use of Laboratory Animals. All efforts were made to minimize suffering and the number of animals used. APP/PS1 mice (4 months old) were divided into 2 groups, APP/PS1 double-transgenic mice with and without SFN treatment (AD+SFN, AD). Wild-type littermates were correspondingly divided into 2 groups, wild-type mice (WT) and wild-type mice with SFN treatment (WT+SFN). Each group had 10 mice (5 males and 5 females), with roughly balanced body weights across the groups. Mice in the WT+SFN and AD+SFN groups were treated with 25 mg/kg SFN (dissolved in distilled water) by a single oral gavage daily, and mice in the AD and WT groups were gavaged with equivalent volumes of distilled water. The dose of SFN was selected based on reports in the literatures (Myzak et al., 2006; Luo et al., 2015) and our previous study (Zhang et al., 2015). Body weight was measured once a week. After 5 months, behavioral tests of mice were performed, and the mice were sacrificed. Their brains were collected, weighed and divided into halves, one of which was stored in 4% paraformaldehyde (pH 7.4) and the other of which was stored at −80°C for later use.

#### Behavior

The open field and Morris water maze tests were performed as we have described in detail elsewhere (Zhang et al., 2014). Briefly, in the open field test, each mouse was placed in the center of a circular open field apparatus (120 cm in diameter and 50 cm high) divided into 25 evenly distributed (central, intermediary, and peripheral) transparent areas and allowed to explore for 3 min. Behavioral parameters, including immobility time in the central area (latency), times of cross areas, and times of upright were measured. After each trial, the floor was cleaned with a damp cloth and dried. Morris water maze test designed to assess spatial learning and memory, was conducted in a pool (120 cm in diameter) filled with water opacified with milk powder maintained at approximately 22 ± 1°C. An escape platform (8 cm in diameter) was placed 1 cm below the water surface. The procedure included escape training trials (days 1–4) and a probe trial (day 5). In the training phase, each mouse was given four trials each day with a 60 s inter-trial interval. The time that elapsed from release to climbing on the hidden platform was recorded as the escape latency for each trial. For the probe trial on day 5, each mouse was again placed in the pool and allowed to swim freely in the water tank without the platform for 60 s; the number of passes through the region of the platform location was recorded.

#### Immunohistochemistry

The hemispheres that were placed in 4% paraformaldehyde (pH 7.4), as mentioned above, were left in paraformaldehyde for 3 days at 4°C, dehydrated in a graded series of alcohols, made transparent with xylene, and then embedded in paraffin. Five-micron-thick serial coronal sections (2.5–3.5 mm posterior of bregma) were cut. Three sections from each mouse, separated serially by 200–300 μm, were used for anti-Aβ_1–42_ immunohistochemistry (Zhang et al., 2015). The selected sections were baked, and then dewaxed in xylene and dehydrated in an alcohol row, subsequently, washed them three times in phosphate-buffered saline (PBS; pH 7.2). The selected sections were incubated in 3% hydrogen peroxide for 10 min and 10% normal goat serum at 37°C for 30 min to block endogenous peroxidase activity and non-specific protein binding, respectively. Sections were incubated with primary rabbit anti-Aβ_1–42_ polyclonal antibody (see Table 1) at 4°C overnight and, subsequently, incubated with goat anti-rabbit immunoglobulin G secondary antibody for 30 min. The sections were then incubated with horseradish peroxide avidinbiotin complex for another 15 min and the reaction products were visualized with diaminobenzidine chromogen solution. Aβ-immunopositive plaques in the whole cerebral cortex of selected sections were observed and counted under an optical microscope. An average of the number of Aβ plaque counts across the three sections was obtained for each animal (*n* = 10 per experimental group).

#### Quantitative reverse transcriptase (qRT)-polymerase chain reaction (PCR) assay of cortex samples

Total mRNA was isolated from thawed cerebral cortex with SV Total RNA Isolation System kit (Promega Corporation, Madison, Wisconsin). Reverse transcription was carried out for each sample using the Prime-Script RT-PCR System kit (TaKaRa Dalian Biotechnology, Dalian, China). The resulting complementary DNAs were used as templates for Real-Time PCR in the ABI 7500 Real-Time PCR system (Applied Biosystems, Inc., Carlsbad, CA) with SYBR Premix Ex Taq Mix (TaKaRa Dalian). The PCR was performed as reported elsewhere (Zhang et al., 2015). The primers were synthesized and purified by TaKaRa Dalian with the following sequences: mus p75NTR, forward: 5′-CCTTGGGGTCACAGATGATG-3′, reverse: 5′-GTTGGGGAGGTTAGTTCCAGAT-3′ (175 bp product); mus HDAC1, forward: 5′-GACCGGTTAGGTTGCTTCAA-3′, reverse: 5′-AACATTCCGGATGGTGTAGC-3′ (120 bp product); mus HDAC2, forward: 5′-GCCAAGTCAGAACAACTCAGC-3′, reverse: 5′-GTCCTCAAACAGGGAAGGTT-3′ (104 bp product); mus HDAC3, forward: 5′-ATCCGCCAGACAATCTTTGA-3′, reverse: 5′-CTCGGGACCTCTCTCTTCAG-3′ (132 bp product); β-actin forward: 5′-CATCCGTAAAGACCTCTATGCCAAC-3′, reverse: 5′-ATGGAGCCACCGATCCACA-3′ (171 bp product). Absolute values from each sample were normalized to β-actin (constitutive gene) mRNA as a reference standard (*n* = 10). Data were expressed by comparative C_T_ method (also known as the 2^−ΔΔCT^ method).

#### Western blot analysis of cortex samples

Thawed cerebral cortex samples were homogenized directly in RIPA buffer containing 0.1% protease inhibitor (Amerso, USA). The lysates were centrifuged at 13,000 rpm for 10 min at 4°C, and the supernatants were used for protein analyses. We determined protein concentrations in the supernatants using the Bradford method with Coomassie Brilliant Blue (CBB G-250) and bovine serum albumin as a standard. The lysates were mixed with β-mercaptoethanol (5%) and bromophenol blue (0.02%), and boiled for 5 min to denature the proteins. Equal amounts of soluble protein were separated by sodium dodecyl sulfate polyacrylamide gel electrophoresis and transferred onto a polyvinylidene fluoride membrane. After 1-h pretreatment in blocking with blocking buffer, the membrane was incubated with rabbit anti-p75NTR, anti-Ace-H3K9, anti-Ace-H4K12, anti-Histone H3.1, anti-HDAC1, anti-HDAC2, anti-HDAC3, or anti-β-actin antibody (see Table 1) overnight at 4°C, followed by goat anti-rabbit IgG secondary antibody (1:8000) for 2 h. Proteins were detected by adding ECL reagent and exposure to a Gel Image System Ver. 4.00 (USA). Band sizes were quantified using the Image J software (Wayne Rasband, National Institutes of Health, Bethesda, MD) (*n* = 10). Sample loading was normalized relative to β-actin or Histone H3.1 as a reference standard.

### *In vitro* study

#### Cell culture

Human SH-SY5Y neuroblastoma cells (The Chinese academy of sciences cell bank, KCB2006107YJ, Kunming, China) were cultured in DMEM/F12 (1:1) media with 10% fetal bovine serum, 100 U/ml penicillin, and 100 μg/ml streptomycin. Cultures were kept in an incubator at 37°C with 5% CO_2_, and cells were passaged with 0.25% trypsin twice a week upon reaching 90% confluence.

#### Cell viability assay

Cell viability was assessed by MTT [(3, 4, 5-dimethylthiazol-2-yl)-2–5-diphenyltetrazolium bromide] assay. Briefly, cells were seeded in 96-well culture microplates at a density of 8 × 10^4^/cm^2^ in 100 μl of antibiotic-free normal growth medium and incubated for 24 h. Cells were incubated with (i.e., pretreatment) or without SFN (final concentration 2 μM). After 3 h, cells were exposed to Aβ_25−35_ [final concentration, 0 μM (blank control), 10, 20, 40, and 80 μM) for 24 h. Then 10 μl of MTT solution (5 μg/ml) was added to each well and incubated for 3 h. Subsequently, the resultant purple MTT-formazan crystals were dissolved by adding 100 μl DMSO to each well. The absorbance of the samples was measured with a Microplate Reader (Biotek, USA) at 490 nm. This experiment was performed in duplicate and repeated three times.

#### qRT-PCR and western blot analyses of cells

Cells were seeded in six-well culture microplates at a density of 1 × 10^5^ cells/well in 2 ml of antibiotic-free normal growth medium, and incubated for 24 h with (SFN and SFN+Aβ groups) or without (control and Aβ groups) inclusion of SFN (final concentration 2 μM); the SFN concentration was selected based on previously published results (Chen et al., 2013; Lee et al., 2013). Three hours later, Aβ_25−35_ (final concentration 20 μM) was mixed quickly into the cultures in the group Aβ and SFN+Aβ groups, and the cells were cultured for an additional 24 h. Subsequently, the cells were collected and submitted to mRNA and protein expression assays by qRT-PCR and western blot, as described above. Specifically, levels of p75NTR, HDAC1, HDAC2, and HDAC3 mRNA in cells were analyzed. The primers were synthesized and purified with the following sequences, homo p75NTR forward: 5′-CCCTGTCTATTGCTCCATCC-3′, reverse: 5′-GTTGGCTCCTTGCTTGTTCT-3′ (103 bp product); homo HDAC1 forward: 5′-ACGACGGGGATGTTGGAAAT-3′, reverse: 5′-TGGCTTTGTGAGGGCGATAG-3′ (135 bp product); homo HDAC2 forward: 5′-AGGTTGAAGCCATTCTCCTG-3′, reverse: 5′-ATCCCAGCACTTTGGAAGG-3′ (179 bp product); homo HDAC3 forward: 5′-GAGGGATGAACGGGTAGACA-3′, reverse: 5′-CAGGTGTTAGGGAGCCAGAG-3′ (137 bp product); β-actin forward: 5′-CATCCGTAAAGACCTCTATGCCAAC-3′, reverse: 5′-ATGGAGCCACCGATCCACA-3′ (171 bp product). Levels of p75NTR, Ace-H3K9, Ace-H4K12, Histone H3.1, HDAC1, HDAC2, and HDAC3 proteins were analyzed with the corresponding primary antibodies (see Table 1). This experiment was performed in duplicate and repeated three times.

#### Small interfering RNA (siRNA)

HDAC1, HDAC2, and HDAC3 siRNA duplex (Guangzhou RIBOBIO CO, LTD) were used to interfere with endogenous HDAC1, HDAC2 and HDAC3 mRNA levels, respectively. Cells were seeded in six-well culture microplates at a density of 1 × 10^5^ cells/well in 2 ml of antibiotic-free normal growth medium. The following day, cells were transfected with 50 nM siRNA duplex and 12 μl of siRNA transfection reagent (Guangzhou RIBOBIO CO, LTD) mixed in DMEM/F12 (1:1) media with 1% fetal bovine serum. The transfected cells were incubated at 37°C for 24 h. Untreated cells and non-specific siRNA (scrambled siRNA; Guangzhou RIBOBIO CO, LTD) were used as controls. The interference efficiency was evaluated by qRT-PCR and western blot analyses. In addition, the expression levels of p75NTR mRNA and protein, and Ace-H3K9 and Ace-H4K12 levels were investigated by the above methods. This experiment was performed in duplicate and repeated three times.

### Statistical analyses

All data are presented as means ± standard deviations (SDs). Group variance was determined with one-way analyses of variance (ANOVAs) followed by Fisher's least significant difference (LSD) multiple comparison *post hoc* tests in SPSS 13.0 software for Windows (version 13.0; SPSS, Chicago, Illinois), except for Morris water maze escape latency data, which were analyzed with a two-way repeated measures ANOVA. Probability values < 0.05 were considered statistically significant.

## Results

### *In vivo* study

#### Sign, body weight, and brain-body weight ratio

During the treatment period, no significant signs of toxicity were observed in the mice. There were no significant differences (*p* > 0.05) in body weight or brain-body weight ratio among the groups (data not reported).

#### SFN ameliorated the cognitive dysfunction of AD model mice

**Figure 1:**
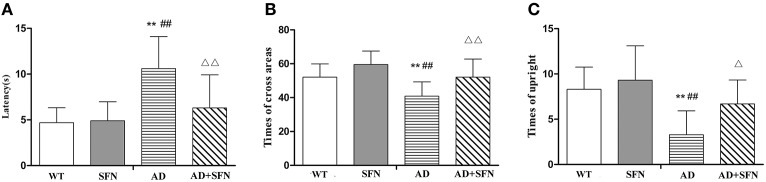
**Analysis of mice behavior in the open field test**. Open field test was used to detect self-independent exploring ability and environmental adaptability. A circular open field consisted of 25 transparent areas at the peripheral, central, and intermediary of the arena. Mice were placed in the center of the open field and allowed to explore for 3 min. **(A)** Times of cross areas. **(B)** Times of upright. **(C)** Immobility time in the central area (latency). (*n* = 10; mean ± SD; One-way ANOVA followed by LSD multiple comparison tests; ^**^p < 0.01 vs. WT group, ^*##*^*p* < 0.01 vs. WT+SFN group, Δ*p* < 0.05 and ΔΔ*p* < 0.01 vs. AD group).

Cognitive deficits are associated with the clinical symptomatology of AD. Open field test was first performed to detect self-independent exploring ability and environmental adaptability. Compared with WT mice with or without SFN treatment, there were significant decreases (*p* < 0.01) in times of cross areas (Figure 1A) and upright (Figure 1B), and a significantly longer latency (Figure 1C) (*p* < 0.01) in the central area in AD model mice. In addition, compared with AD model mice, obvious increases (*p* < 0.05; *p* < 0.01) in times of cross areas and upright, and a significantly shorter latency (*p* < 0.01) in the central area were found in SFN-treated AD model mice.

**Figure 2:**
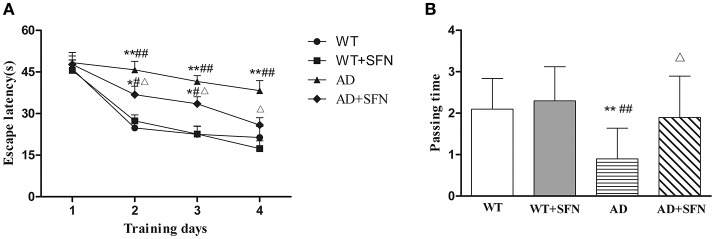
**Analysis of mice behavior in Morris water maze**. Morris water maze was conducted to test spatial learning and memory in a circular pool filled with milk-opacified water. The procedure included escape trials (4 days) and a probetrial (1 day). In escape trials, an escape platform (8 cm in diameter) was placed 1 cm below the water surface. Each mouse was given 4 trials with 60 s intertrial interval per day. In the probe trial, mice swam freely in the water tank without the platform for 60 s. **(A)** Escape latency to the hidden platform in escape trials. **(B)** Pass through the region of the original platform (passing times) in the probe trial. (*n* = 10; mean ± SD; two-way ANOVA with repeated measures for escape latency and one-way ANOVA for passing time both followed by LSD multiple comparison tests; ^*^p < 0.05 and ^**^p < 0.01 vs. WT group, #*p* < 0.05 and ^*##*^*p* < 0.01 vs. WT+SFN group, Δ*p* < 0.05 vs. AD group).

Next, the Morris water maze was conducted to test spatial learning and memory. During Morris water maze acquisition training (Figure 2A), compared with WT mice with or without SFN treatment, AD model mice had longer (*p* < 0.01) escape latencies on days 2–4 and SFN-treated AD model mice had longer (*p* < 0.05) escape latencies on days 2 and 3, but notably not on day 4. The escape latencies for the AD group were longer than those of the AD+SFN group on days 2–4 (*p* < 0.05). In the probe trial (Figure 2B), the passing times significantly reduced (*p* < 0.01) in AD model mice compared with WT mice with or without SFN treatment. However, compared with AD model mice, a remarkable increase (*p* < 0.05) in passing times was found in SFN-treated AD model mice.

#### SFN protected against the increment of Aβ plaques in the cerebral cortex of AD model mice

**Figure 3:**
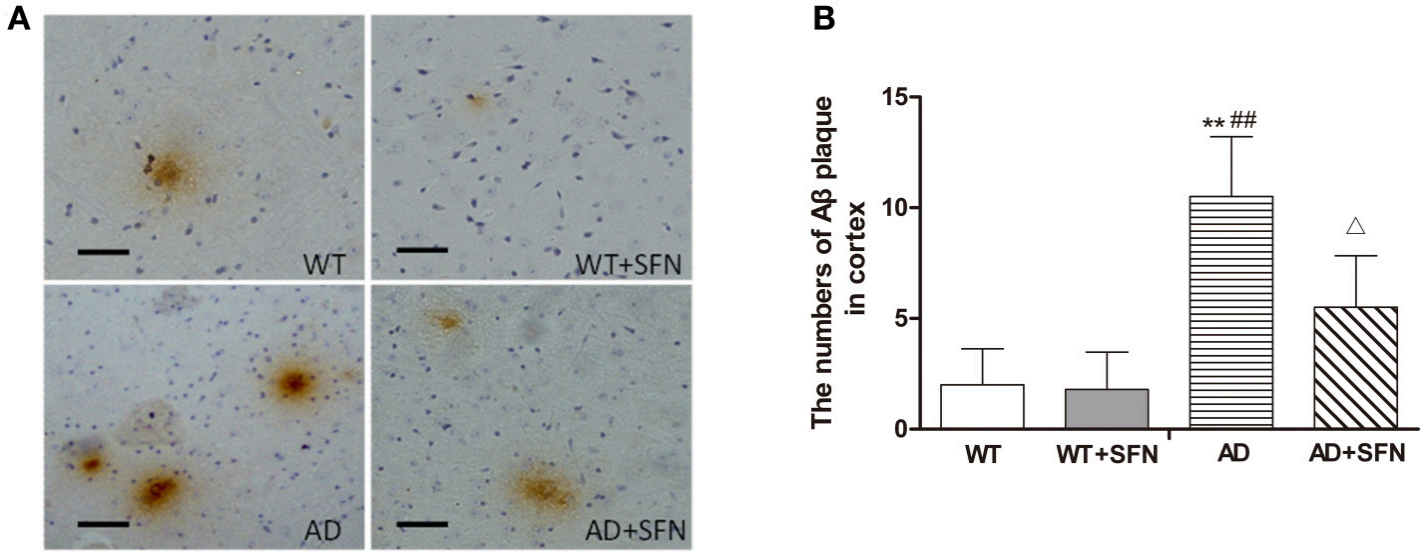
**SFN protected against the increment of Aβ plaques in the cerebral cortex of AD model mice. (A)** Brown immunoreactive plaques imply the Aβ deposition in the cerebral cortex of mice (bars = 10 μm). **(B)** The average number of Aβ plaques in the whole cerebral cortex from each section. (*n* = 10; mean ± SD; One-way ANOVA followed by LSD multiple comparison tests; ^**^p < 0.01 vs. WT group, ^*##*^*p* < 0.01 vs. WT + SFN group, Δ*p* < 0.05 vs. AD group; magnify 400 ×).

To investigate whether SFN protects against the increment of Aβ plaques, the number of Aβ plaques in the cerebral cortex was investigated by immunohistochemistry (Figure 3). Brown plaques imply the localization of Aβ immunoreactivity in mice brains. The numbers of Aβ immunopositive plaques were markedly elevated (*p* < 0.01) in the cerebral cortex of AD model mice compared with WT mice with or without SFN treatment. Moreover, compared with AD model mice, the numbers of Aβ immunopositive plaques in the cerebral cortex were significantly decreased (*p* < 0.05) in AD model mice with SFN treatment.

#### SFN protected against the decreased expression of p75NTR mRNA and protein in the cerebral cortex of AD model mice

**Figure 4:**
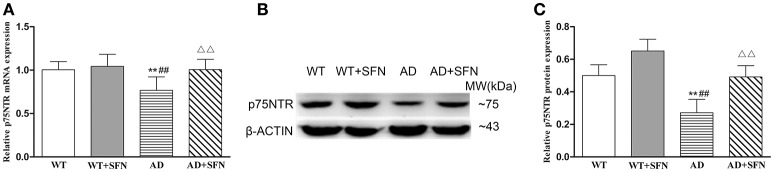
**SFN protected against the decreased expression of p75NTR mRNA and protein in the cerebral cortex of AD model mice**. The relative expression of p75NTR mRNA (β-actin as a reference standard) **(A)** and protein **(B**,**C)** were analyzed by qRT-PCR and Western blot, respectively. (*n* = 10; mean ± SD; One-way ANOVA followed by LSD multiple comparison tests; ^**^p < 0.01 vs. WT group, ^*##*^*p* < 0.01 vs. WT + SFN group, ΔΔ*p* < 0.01 vs. AD group).

To explore the possible mechanisms responsible for the role of SFN in AD, we examined the mRNA (Figure 4A) and protein (Figures 4B,C) expression of p75NTR in the cerebral cortex by qRT-PCR and western blot, respectively. In the cerebral cortex of AD model mice, p75NTR mRNA and protein expression levels were significantly decreased (*p* < 0.01) relative to the levels observed in WT mice with or without SFN treatment. Meanwhile, SFN-treated AD model mice had increased significantly p75NTR mRNA and protein expression levels in the cerebral cortex (*p* < 0.01) compared with AD model mice.

#### SFN protected against the decreased levels of Ace-H3K9 and Ace-H4K12 in the cerebral cortex of AD model mice

**Figure 5:**
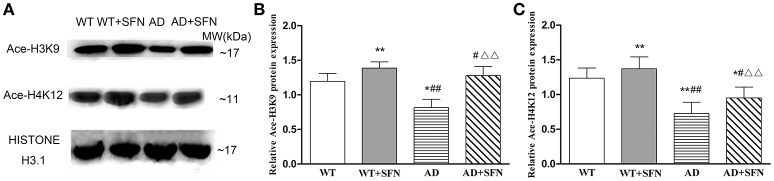
**SFN prevented from decreasing the levels of Ace-H3K9 and Ace-H4K12 protein in the cerebral cortex of AD model**. Western blot was used to analyze the relative protein expression of Ace-H3K9 **(A,C)** and Ace-H4K12 (**B,C**). (*n* = 10; mean ± SD; One-way ANOVA followed by LSD multiple comparison tests; ^*^p < 0.05, ^**^p < 0.01 vs. WT group, ^#^*p* < 0.05, ^*##*^*p* < 0.01 vs. WT + SFN group, ΔΔ*p* < 0.01 vs. AD group).

SFN is a pan-HDAC inhibitor. We investigated the effect of SFN on the level of histone acetylation in the cerebral cortex of AD model mice (Figure 5). Compared with WT mice, the levels of Ace-H3K9 and Ace-H4K12 is significantly decreased (*p* < 0.05; *p* < 0.01) in the cerebral cortex of AD model mice, and obviously increased (*p* < 0.01) in WT mice with SFN treatment. Additionally, increased acetylation of histone H3 and H4 at lysine K9 and K12 respectively levels were observed in the cerebral cortex of AD model mice treated with SFN compared with vehicle (*p* < 0.01).

#### SFN prevented from increasing the expression of HDAC1, HDAC2, and HDAC3 mRNA and protein in the cerebral cortex of AD model mice

**Figure 6:**
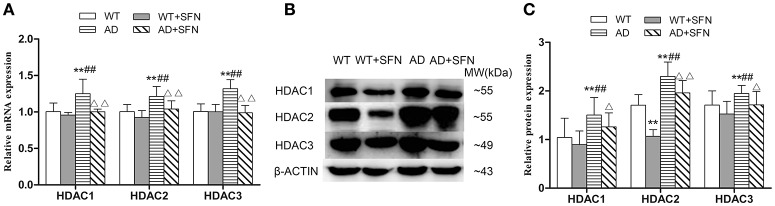
**SFN prevented from increasing the expression of HDACs mRNA and protein in the cerebral cortex of AD model mice**. The relative expression of HDAC1, HDAC2 and HDAC3 mRNA (β-actin as a reference standard) **(A)** and protein **(B,C)** were analyzed by qRT-PCR and Western blot, respectively. (*n* = 10; mean ± SD; One-way ANOVA followed by LSD multiple comparison tests; ^**^p < 0.01 vs. WT group, ^*##*^*p* < 0.01 vs. WT + SFN group, Δ*p* < 0.05, ΔΔ*p* < 0.01 vs. AD group).

To investigate whether HDAC subtype is regulated by SFN in the cerebral cortex of AD model mice, we examined the mRNA and protein expression of HDAC1, HDAC2 and HDAC3. Our qRT-PCR (Figure 6A) and western blot (Figures 6B,C) analyses showed that HDAC1, HDAC2, and HDAC3 mRNA and protein expression levels in the cerebral cortex of AD model mice were significantly increased (*p* < 0.01) compared with levels observed in WT mice with or without SFN treatment, and obviously decreased (*p* < 0.05; *p* < 0.01) in AD model mice treated with SFN relative to the levels observed in AD model mice.

### *In vitro* study

To further investigate whether SFN up-regulates p75NTR expression through regulating the levels of histone acetylation, we performed the experiments *in vitro* using AD model cells (Aβ-induced SH-SY5Y cells) and to in-depth explore the possible mechanism with siRNA knock down of HDACs in SH-SY5Y cells.

#### SFN protected SH-SY5Y cells against Aβ-induced decrease of viability

**Figure 7:**
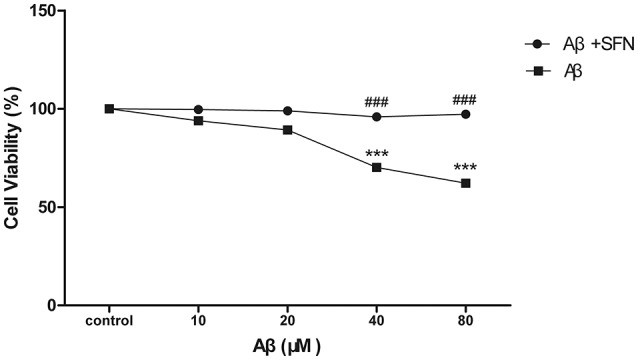
**SFN protected SH-SY5Y cells against Aβ-induced decrease of viability**. Analysis of cell viability was performed by MTT. Before exposure to Aβ_25−35_, cells in SFN+Aβ group were pre-treated with SFN (final concentration of 2μM) for 3 h. Both Aβ_25−35_ and SFN are dissolved in water. Cells in control group were not treated with SFN or Aβ_25−35_. (*n* = 6; mean ± SD; One-way ANOVA followed by LSD multiple comparison tests; ^***^p < 0.001 vs. control group, ###*p* < 0.001 vs. Aβ group).

Cell viability was performed by MTT assay. After being exposed to different concentrations of Aβ (10–80 μM), the cell viabilities were decrease Aβ-induced in a dose-dependent manner. Compared with control, significant decreases (*p* < 0.001) were found in cells treated with Aβ at concentrations of 40 and 80 μM. SFN pretreatment remarkably inhibited the decreases of cell viabilities induced by Aβ at concentrations of 40 and 80 μM (*p* < 0.001; Figure 7).

#### SFN protected against the decreased expression of p75NTR mRNA and protein in Aβ-treated SH-SY5Y cells

**Figure 8:**
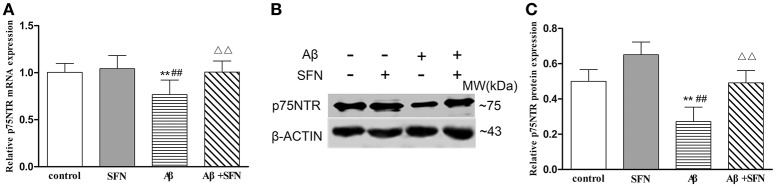
**SFN protected against the decreased expression of p75NTR mRNA and protein in Aβ-treated SH-SY5Y cells**. The relative expression of p75NTR mRNA (β-actin as a reference standard) **(A)** and protein **(B,C)** were analyzed by qRT-PCR and Western blot, respectively. (*n* = 6; mean ± SD; One-way ANOVA followed by LSD multiple comparison tests; ^**^p < 0.01 vs. control group, ^*##*^*p* < 0.01 vs. SFN group, ΔΔ*p* < 0.01 vs. Aβ group).

Cells exposed to 20 μM Aβ had decreased p75NTR mRNA (Figure 8A) and protein (Figures 8B,C) expression levels compared with those observed in non-Aβ exposed control cells, with or without SFN pretreatment (*p* < 0.01). Meanwhile, SFN-pretreated cells exposed to Aβ had increased p75NTR mRNA and protein levels compared with the levels observed in non-pretreated Aβ-exposed cells (*p* < 0.01).

#### SFN prevented from decreasing the expression of Ace-H3K9 and Ace-H4K12 protein in Aβ-treated SH-SY5Y cells

**Figure 9:**
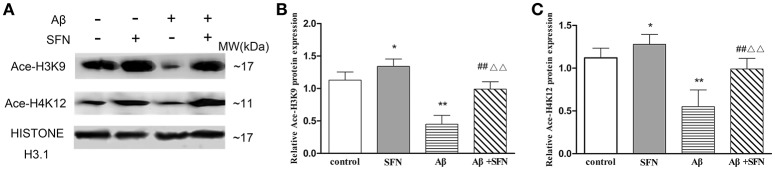
**SFN prevented from decreasing the levels of Ace-H3K9 and Ace-H4K12 protein in Aβ-treated SH-SY5Y cells**. Western blot was used to analyze the relative protein expression of Ace-H3K9 **(A,C)** and Ace-H4K12 **(B,C)**. (*n* = 6; mean ± SD; One-way ANOVA followed by LSD multiple comparison tests; ^*^p < 0.05, ^**^p < 0.01 vs. control group, ^*##*^*p* < 0.01 vs. SFN group, ΔΔ*p* < 0.01 vs. Aβ group).

As shown in Figure 9, compared with levels in control cells, Ace-H3K9 and Ace-H4K12 levels were significantly decreased (*p* < 0.01) in cells exposed Aβ, and increased (*p* < 0.05) in cells pretreated with SFN. Moreover, compared with cells exposed to Aβ without SFN, we observed increased (*p* < 0.01) Ace-H3K9 and Ace-H4K12 protein levels in Aβ-exposed cells in the presence of SFN.

#### SFN inhibited the increased expression of HDAC1, HDAC2, and HDAC3 mRNA and protein in Aβ-treated SH-SY5Y cells

**Figure 10:**
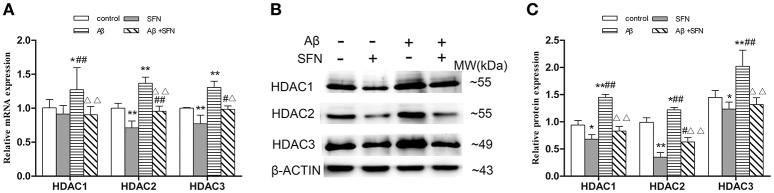
**SFN inhibited the increased expression of HDACs mRNA and protein in Aβ-treated SH-SY5Y cells**. The relative expression of HDAC1, HDAC2 and HDAC3 mRNA (β-actin as a reference standard) **(A)** and protein **(B,C)** were analyzed by qRT-PCR and Western blot, respectively. (*n* = 6; mean ± SD; One-way ANOVA followed by LSD multiple comparison tests; ^*^p < 0.05, ^**^p < 0.01 vs. control group, ^#^*p* < 0.05, ^*##*^*p* < 0.01 vs. SFN group, Δ*p* < 0.01, ΔΔ*p* < 0.01 vs. Aβ group).

The levels of HDACs mRNA and protein expression *in vitro* are shown in Figures 10A–C, respectively. Compared with the expression levels observed in control, HDAC1, HDAC2, and HDAC3 mRNA and protein expression levels were increased (*p* < 0.05; *p* < 0.01) in cells exposed to Aβ, and decreased (*p* < 0.05; *p* < 0.01) in cells pretreated with SFN. HDAC1, HDAC2, and HDAC3 mRNA and protein levels were decreased (*p* < 0.05; *p* < 0.01) in cells that were pretreated with SFN and exposed to Aβ compared with cells only exposed to Aβ without SFN.

#### Up-regulation of p75NTR expression in HDAC1- and HDAC3- but not HDAC2-silenced SH-SY5Y cells

**Figure 11:**
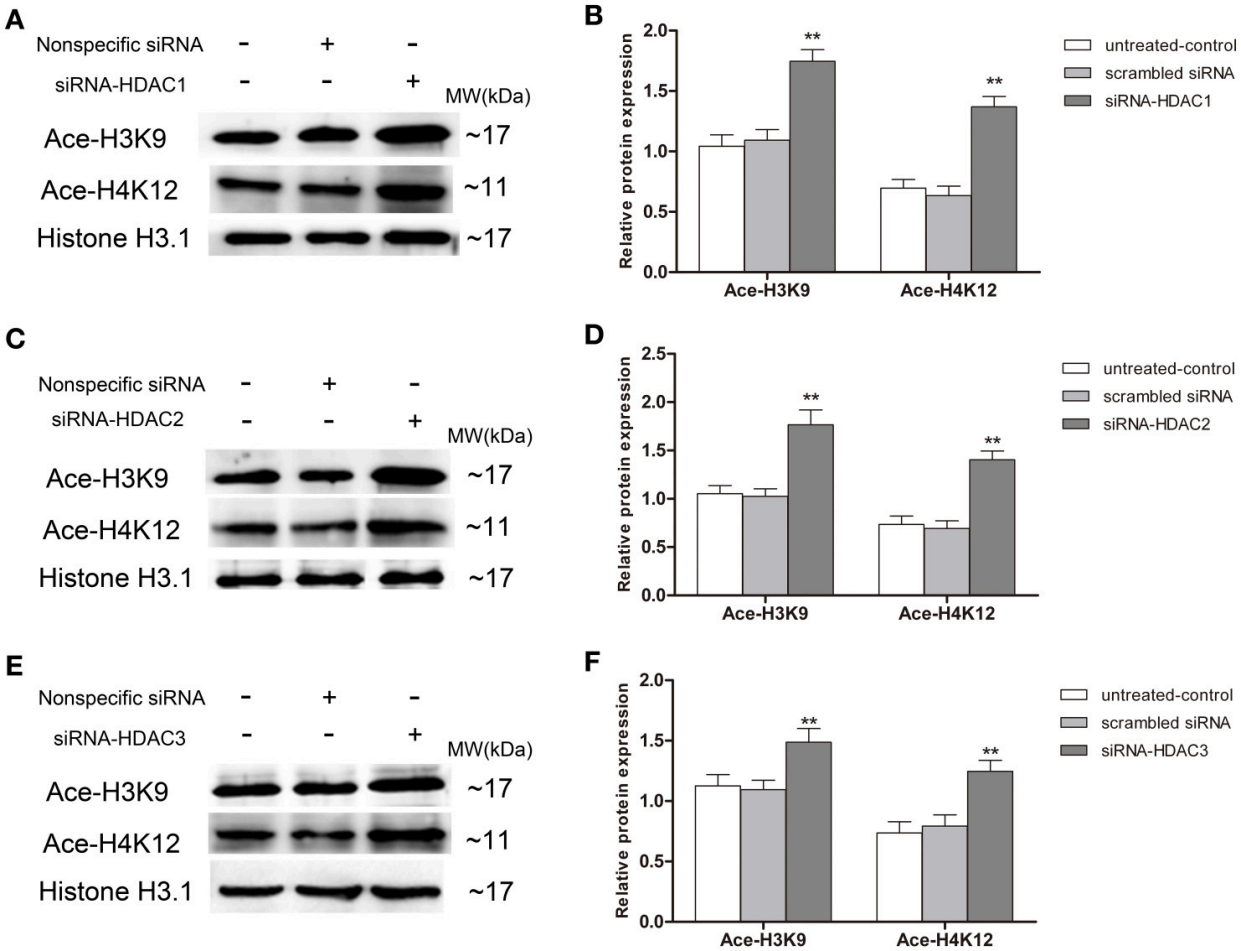
**Increment of Ace-H3K9 and Ace-H4K12 levels in HDAC1-, HDAC2- or HDAC3-silenced SH-SY5Y cells**. siRNA duplex was used to interfere with endogenous HDAC1, HDAC2 or HDAC3 mRNA expression (untreated cells and non-specific siRNA as controls). The relative levels of Ace-H3K9 and Ace-H4K12 were analyzed by Western blot in HDAC1-silenced **(A,B)**, HDAC2-silenced **(C,D)** or HDAC3-silenced **(E,F)** cells. (*n* = 6; mean ± SD; One-way ANOVA followed by LSD multiple comparison tests; ^**^p < 0.01 vs. control groups).

**Figure 12:**
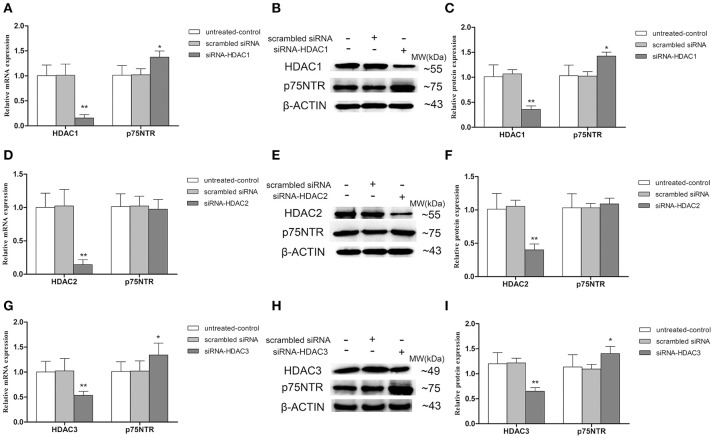
**Up-regulation of p75NTR expression in HDAC1- or HDAC3- but not HDAC2-silenced SH-SY5Y cells**. siRNA duplex was used to interfere with endogenous HDAC1, HDAC2 or HDAC3 mRNA expression (untreated cells and non-specific siRNA as controls). The relative expression levels of p75NTR mRNA and protein were analyzed by qRT-PCR (β-actin as a reference standard) and Western blot, respectively, in HDAC1-silenced **(A–C)**, HDAC2-silenced **(D–F)** or HDAC3-silenced **(G–I)** cells. (*n* = 6; mean ± SD; One-way ANOVA followed by LSD multiple comparison tests; ^*^p < 0.05, ^**^p < 0.01 vs. control groups).

To further determinate which HDAC subtype regulates p75NTR expression, siRNA duplex was used to interfere with endogenous HDAC1, HDAC2, or HDAC3 mRNA expression. As illustrated in Figure 11, levels of Ace-H3K9 and Ace-H4K12 was significantly increased in HDAC1 (Figures 11A,B), HDAC2 (Figures 11C,D) or HDAC3 (Figures 11E,F) knock-down cells compared with the levels in controls (*p* < 0.01). Furthermore, we found that expression of p75NTR mRNA and protein was increased in HDAC1 (Figures 12A–C) or HDAC3 (Figures 12G–I) knock-down cells compared with p75NTR levels in controls (*p* < 0.05). However, no differences in p75NTR mRNA and protein expression were found between HDAC2 (Figures 12D–F) knock-down cells and controls (*p* > 0.05).

## Discussion

Cognitive dysfunction, especially memory loss, is the prominent and earliest clinical manifestation in AD patients, running through the whole course of the disease. Our behavioral results demonstrated impaired Morris water maze spatial memory acquisition and retrieval as well as compromised exploratory behavior and environmental adaptability, represented by open field behavior, in AD model mice. In recent years, the phytochemical SFN has attracted attention as a possible pharmacotherapeutic agent aimed at ameliorating cognitive dysfunction. Orally administered SFN is absorbed rapidly, resulting in high absolute bioavailability and crosses the blood-brain barrier readily (Hanlon et al., 2008; Benedict et al., 2012; Dominguez-Perles et al., 2014). Our present findings of behavioral benefits of SFN are consistent with our previous work demonstrating that SFN (25 mg/kg) ameliorated cognitive deficits in mice induced by combined D-galactose and aluminum administration (Zhang et al., 2014, 2015). Furthermore, it has been reported that SFN at 5 mg/kg and 25 mg/kg doses prevented cognitive impairment related to traumatic brain injury and diabetes respectively (Dash et al., 2009; Wang et al., 2016). Lines of evidence indicate that behavioral dysfunction is mainly associated with Aβ deposition in the brain (Hardy and Selkoe, 2002). Consistent with a previous study (Zhang et al., 2015), SFN was found in this study to play a role in protecting against the increment of Aβ deposition in the brains of AD model mice. Furthermore, Park et al found previously that pretreatment of cells with SFN provided significant protection against Aβ exposure in Neuro 2A cells (Park et al., 2009). The results from this study also showed that SFN possessed anti-Aβ effects against reduced cell vitality in SH-SY5Y cells in the presence of Aβ to mimic AD *in vitro*. These suggest that SFN may have a protective effect for cognitive function and neurons through reducing Aβ deposition and/or against Aβ toxicity.

The broadly expressed p75NTR protein has been reported to inhibit Aβ deposition and to be neuroprotective (Yano and Chao, 2000; Wang et al., 2011). The present observations of decreased expression of p75NTR in both the cerebral cortex of AD model mice and SH-SY5Y cells treated with Aβ are consistent with the findings of many human and animal studies (Kordower et al., 1989; Arendt et al., 1997; Salehi et al., 2000). However, Chakravarthy et al. found that p75NTR expression was increased in AD model mouse brains and AD model cells (Chakravarthy et al., 2010), This discrepancy could be due to methodological differences, such as differences in the brain regions investigated, Aβ dosages, and the types of measurements taken (Zeng et al., 2011). Meanwhile, the expression of p75NTR mRNA and protein, in this study, were up-regulated by SFN in the cerebral cortex of AD model mice and Aβ-treated SH-SY5Y cells. Our findings suggest that SFN may protect against AD through up-regulation of p75NTR and, thereby, inhibition of Aβ deposition and/or toxicity. In addition to its binding affinity for Aβ p75NTR also binds several neurotrophic factors, including brain-derived neurotrophic factor (BDNF) and nerve growth factor. The findings of Kim et al. suggest that epigenetic enhancement of neuronal BDNF expression by SFN maybe neuroprotective, and thus that that p75NTR, acting as a neurotrophin receptor, may help protect against AD (Kim et al., 2017).

Our findings that pan-HDAC inhibitor SFN increased Ace-H3K9 and Ace-H4K12 levels in the cerebral cortex of wild type mice and in SH-SY5Y cells, and countered the opposite effects by Aβ, provide clues about the mechanism of action of SFN with respect to AD pathology and behavioral symptoms. Other HDAC inhibitors (romidepsin and TSA) have been reported to induce p75NTR expression in neuroblastoma tumor cells (Panicker et al., 2010; Iraci et al., 2011). However, the HDAC inhibitor LB205 suppressed p75NTR expression in a rodent traumatic brain injury model (Lu et al., 2013). The reasons for these apparently inconsistent results are not yet known, but possible explanatory factors include the use of different drugs with distinct HDAC subtype-dependent influences and the use of different model animals or cells.

Some HDAC inhibitors have been reported to decrease the protein but not mRNA expression levels of HDAC subtypes, such as the effects of SAHA on HDAC2 and HDAC4 (Mielcarek et al., 2011). In this study, SFN was found to decrease not only protein but also mRNA expression levels of HDAC1, HDAC2, and HDAC3 in the cerebral cortex of wild type mice and in SH-SY5Y cells. Furthermore, our results showed that SFN alleviated the increased expression of HDAC1, HDAC2, and HDAC3 in the cerebral cortex of AD model mice and in Aβ-induced SH-SY5Y cells. In addition, we found that the levels of Ace-H3K9 and Ace-H4K12 were increased in HDAC1-, HDAC2- or HDAC3-silenced cells. Our finding that the expression of p75NTR was elevated in HDAC1-silenced and HDAC3-silenced but not HDAC2-silenced SH-SY5Y cells, indicates that SFN-induced up-regulation of p75NTR may be mediated, at least in part, by reducing HDAC1 and HDAC3 expression, but not HDAC2. A prior report showing that HDAC1 regulates p75NTR expression by binding to its promoter region (Iraci et al., 2011) is consistent with this inference.

This study has several limitations. Firstly, it remains to be confirmed experimentally whether reduction of HDAC3 expression increases Ace-H3K9 and Ace-H4K12 levels in the p75NTR promoter region. Secondly, further study is needed to examine the potential involvement of other SFN-modulated HDACs (HDAC6 and HDAC8) in SFN effects on AD pathology and symptoms. Thirdly, immunohistochemistry is a semiquantitative method and the results related to a reduction of Aβ plaques by immunohistochemistry should be confirmed by more quantitative assays i.e., ELISA. Finally, because we administered SFN as a pretreatment ahead of Aβ plaque formation and exposure, further studies are needed to determine whether SFN has beneficial effects after Aβ plaques have already formed.

Taken together, in the present study, SFN was observed to improve cognitive function and to protect against Aβ deposition in AD model mice. Up-regulation of p75NTR, mediated at least in part by reduction of HDAC1 and HDAC3 expression, was implicated as a potential anti-Aβ mechanism of SFN. SAHA has been documented to play a protective role in Huntington's disease through decreasing HDAC 2 and 4 levels (Mielcarek et al., 2011). Thus, the effects of different HDAC inhibitors on various diseases may be mediated through regulating different HDAC types. In addition, other biological functions of SFN, such as anti-oxidation (Zhang et al., 2015), anti-inflammatory (Brandenburg et al., 2010), immunoregulation (Shih et al., 2016), and so on, may also play roles in protecting against AD. In conclusion, the findings support further exploration of SFN as a potential candidate drug for AD therapy and prevention.

## Author contributions

LA conceived and designed the experiments. JZ, RZ, and ZZ performed the experiments. XL and FZ analyzed the data. AX, CJ, and YC contributed reagents and materials. JZ and LA drafted the manuscript. All authors approved the final version to be published.

### Conflict of interest statement

The authors declare that the research was conducted in the absence of any commercial or financial relationships that could be construed as a potential conflict of interest.

## References

[B1] Arendt T. Schindler C. Brückner M. K. Eschrich K. Bigl V. Zedlick D.. (1997). Plastic neuronal remodeling is impaired in patients with Alzheimer's disease carrying apolipoprotein epsilon 4 allele. J. Neurosci. 17, 516–529. 8987775 10.1523/JNEUROSCI.17-02-00516.1997 PMC6573225

[B2] Attems J. Thal D. R. Jellinger K. A. (2012). The relationship between subcortical tau pathology and Alzheimer's disease. Biochem. Soc. Trans. 40, 711–715. 10.1042/BST20120034 22817721

[B3] Benedict A. L. Mountney A. Hurtado A. Bryan K. E. Schnaar R. L. Dinkova-Kostova A. T.. (2012). Neuroprotective effects of sulforaphane after contusive spinal cord injury. J. Neurotrauma 29, 2576–2586. 10.1089/neu.2012.2474 22853439 PMC3495118

[B4] Brandenburg L. O. Kipp M. Lucius R. Pufe T. Wruck C. J. (2010). Sulforaphane suppresses LPS-induced inflammation in primary rat microglia. Inflamm. Res. 59, 443–450. 10.1007/s00011-009-0116-5 19924513

[B5] Chakravarthy B. Gaudet C. Mènard M. Atkinson T. Brown L. Laferla F. M.. (2010). Amyloid-beta peptides stimulate the expression of the p75(NTR) neurotrophin receptor in SHSY5Y human neuroblastoma cells and AD transgenic mice. J. Alzheimer's Dis. 19, 915–925. 10.3233/JAD-2010-1288 20157247

[B6] Chen X. Liu J. Chen S. Y. (2013). Sulforaphane protects against ethanol-induced oxidative stress and apoptosis in neural crest cells by the induction of Nrf2-mediated antioxidant response. Br. J. Pharmacol. 169, 437–448. 10.1111/bph.12133 23425096 PMC3651668

[B7] Chin-Chan M. Navarro-Yepes J. Quintanilla-Vega B. (2015). Environmental pollutants as risk factors for neurodegenerative disorders: Alzheimer and Parkinson diseases. Front. Cell. Neurosci. 9:124. 10.3389/fncel.2015.00124 25914621 PMC4392704

[B8] Dash P. K. Zhao J. Orsi S. A. Zhang M. Moore A. N. (2009). Sulforaphane improves cognitive function administered following traumatic brain injury. Neurosci. Lett. 460, 103–107. 10.1016/j.neulet.2009.04.028 19515491 PMC2700200

[B9] Dashwood R. H. Ho E. (2007). Dietary histone deacetylase inhibitors: from cells to mice to man. Semin. Cancer Biol. 17, 363–369. 10.1016/j.semcancer.2007.04.001 17555985 PMC2737738

[B10] Dominguez-Perles R. Medina S. Moreno D. A. Garcia-Viguera C. Ferreres F. Gil-Izquierdo A. (2014). A new ultra-rapid UHPLC/MS/MS method for assessing glucoraphanin and sulforaphane bioavailability in human urine. Food Chem. 143, 132–138. 10.1016/j.foodchem.2013.07.116 24054222

[B11] Hanlon N. Coldham N. Gielbert A. Kuhnert N. Sauer M. J. King L. J.. (2008). Absolute bioavailability and dose-dependent pharmacokinetic behaviour of dietary doses of the chemopreventive isothiocyanate sulforaphane in rat. Br. J. Nutr. 99, 559–564. 10.1017/S0007114507824093 17868493

[B12] Hardy J. Selkoe D. J. (2002). Medicine - The amyloid hypothesis of Alzheimer's disease: progress and problems on the road to therapeutics. Science 297, 353–356. 10.1126/science.1072994 12130773

[B13] Iraci N. Diolaiti D. Papa A. Porro A. Valli E. Gherardi S.. (2011). A SP1/MIZ1/MYCN repression complex recruits HDAC1 at the TRKA and p75NTR promoters and affects neuroblastoma malignancy by inhibiting the cell response to NGF. Cancer Res. 71, 404–412. 10.1158/0008-5472.CAN-10-2627 21123453

[B14] Kim J. Lee S. Choi B. R. Yang H. Hwang Y. Park J. H.. (2017). Sulforaphane epigenetically enhances neuronal BDNF expression and TrkB signaling pathways. Mol. Nutr. Food Res. 61. 10.1002/mnfr.201600194 27735126

[B15] Kordower J. H. Gash D. M. Bothwell M. Hersh L. Mufson E. J. (1989). Nerve growth factor receptor and choline acetyltransferase remain colocalized in the nucleus basalis (Ch4) of Alzheimer's patients. Neurobiol. Aging 10, 67–74. 10.1016/S0197-4580(89)80013-2 2547171

[B16] Lee C. Park G. H. Lee S. R. Jang J. H. (2013). Attenuation of beta-amyloid-induced oxidative cell death by sulforaphane via activation of NF-E2-related factor 2. Oxid. Med. Cell. Longev. 2013:313510. 10.1155/2013/313510 23864927 PMC3705986

[B17] Lu J. Frerich J. M. Turtzo L. C. Li S. Chiang J. Yang C.. (2013). Histone deacetylase inhibitors are neuroprotective and preserve NGF-mediated cell survival following traumatic brain injury. Proc. Natl. Acad. Sci. U.S.A. 110, 10747–10752. 10.1073/pnas.1308950110 23754423 PMC3696796

[B18] Luo L. Chen Y. Wu D. Shou J. Wang S. Ye J.. (2015). Differential expression patterns of Nqo1, AKR1B8 and Ho-1 in the liver and small intestine of C57BL/6 mice treated with sulforaphane. Data Brief 5, 416–423. 10.1016/j.dib.2015.09.029 26958603 PMC4773386

[B19] Mielcarek M. Benn C. L. Franklin S. A. Smith D. L. Woodman B. Marks P. A.. (2011). SAHA decreases HDAC 2 and 4 levels *in vivo* and improves molecular phenotypes in the R6/2 mouse model of Huntington's disease. PLoS ONE 6:e27746. 10.1371/journal.pone.0027746 22140466 PMC3225376

[B20] Myzak M. C. Dashwood W. M. Orner G. A. Ho E. Dashwood R. H. (2006). Sulforaphane inhibits histone deacetylase *in vivo* and suppresses tumorigenesis in Apc-minus mice. FASEB J. 20, 506–508. 10.1096/fj.05-4785fje 16407454 PMC2373266

[B21] Panicker J. Li Z. McMahon C. Sizer C. Steadman K. Piekarz R.. (2010). Romidepsin (FK228/depsipeptide) controls growth and induces apoptosis in neuroblastoma tumor cells. Cell Cycle 9, 1830–1838. 10.4161/cc.9.9.11543 20404560 PMC6659113

[B22] Park H. M. Kim J. A. Kwak M. K. (2009). Protection against amyloid beta cytotoxicity by sulforaphane: role of the proteasome. Arch. Pharm. Res. 32, 109–115. 10.1007/s12272-009-1124-2 19183883

[B23] Plagg B. Ehrlich D. Kniewallner K. M. Marksteiner J. Humpel C. (2015). Increased acetylation of Histone H4 at Lysine 12 (H4K12) in monocytes of transgenic Alzheimer's mice and in human patients. Curr. Alzheimer Res. 12, 752–760. 10.2174/1567205012666150710114256 26159193 PMC4589156

[B24] Prince M. J. (2015). World Alzheimer Report 2015: The Global Impact of Dementia: an Analysis of Prevalence, Incidence, Cost and Trends.

[B25] Salehi A. Ocampo M. Verhaagen J. Swaab D. F. (2000). P75 neurotrophin receptor in the nucleus basalis of Meynert in relation to age, sex, and Alzheimer's disease. Exp. Neurol. 161, 245–258. 10.1006/exnr.1999.7252 10683291

[B26] Shih Y. L. Wu L. Y. Lee C. H. Chen Y. L. Hsueh S. C. Lu H. F.. (2016). Sulforaphane promotes immune responses in a WEHI3induced leukemia mouse model through enhanced phagocytosis of macrophages and natural killer cell activities *in vivo*. Mol. Med. Rep. 13, 4023–4029. 10.3892/mmr.2016.5028 27035756

[B27] Su Z. Y. Zhang C. Lee J. H. Shu L. Wu T. Y. Khor T. O.. (2014). Requirement and epigenetics reprogramming of Nrf2 in suppression of tumor promoter TPA-induced mouse skin cell transformation by sulforaphane. Cancer Prev. Res. 7, 319–329. 10.1158/1940-6207.CAPR-13-0313-T 24441674

[B28] Walker M. P. LaFerla F. M. Oddo S. S. Brewer G. J. (2013). Reversible epigenetic histone modifications and Bdnf expression in neurons with aging and from a mouse model of Alzheimer's disease. Age (Dordr) 35, 519–531. 10.1007/s11357-011-9375-5 22237558 PMC3636384

[B29] Wang G. Fang H. Zhen Y. Xu G. Tian J. Zhang Y.. (2016). Sulforaphane prevents neuronal apoptosis and memory impairment in diabetic rats. Cell. Physiol. Biochem. 39, 901–907. 10.1159/000447799 27497670

[B30] Wang Y. J. Wang X. Lu J. J. Li Q. X. Gao C. Y. Liu X. H.. (2011). p75NTR regulates Abeta deposition by increasing Abeta production but inhibiting Abeta aggregation with its extracellular domain. J. Neurosci. 31, 2292–2304. 10.1523/JNEUROSCI.2733-10.2011 21307265 PMC6633040

[B31] Xu K. Dai X. L. Huang H. C. Jiang Z. F. (2011). Targeting HDACs: a promising therapy for Alzheimer's disease. Oxid. Med. Cell. Longev. 2011:143269. 10.1155/2011/143269 21941604 PMC3177096

[B32] Yano H. Chao M. V. (2000). Neurotrophin receptor structure and interactions. Pharm. Acta Helv. 74, 253–260. 10.1016/S0031-6865(99)00036-9 10812966

[B33] Yao X. Q. Jiao S. S. Saadipour K. Zeng F. Wang Q. H. Zhu C.. (2015). p75NTR ectodomain is a physiological neuroprotective molecule against amyloid-beta toxicity in the brain of Alzheimer's disease. Mol Psychiatry. 20, 1301–1310. 10.1038/mp.2015.49 25917367 PMC4759103

[B34] Zeng F. Lu J. J. Zhou X. F. Wang Y. J. (2011). Roles of p75NTR in the pathogenesis of Alzheimer's disease: a novel therapeutic target. Biochem. Pharmacol. 82, 1500–1509. 10.1016/j.bcp.2011.06.040 21762680

[B35] Zhang R. Miao Q. W. Zhu C. X. Zhao Y. Liu L. Yang J.. (2015). Sulforaphane ameliorates neurobehavioral deficits and protects the brain from amyloid beta deposits and peroxidation in mice with Alzheimer-like lesions. Am. J. Alzheimer's Dis. Other Demen. 30, 183–191. 10.1177/1533317514542645 25024455 PMC10852928

[B36] Zhang R. Zhang J. Fang L. Li X. Zhao Y. Shi W.. (2014). Neuroprotective effects of sulforaphane on cholinergic neurons in mice with Alzheimer's disease-like lesions. Int. J. Mol. Sci. 15, 14396–14410. 10.3390/ijms150814396 25196440 PMC4159858

